# Varying Social Experiences in Adulthood Do Not Differentially Affect Anxiety-Like Behavior But Stress Hormone Levels

**DOI:** 10.3389/fnbeh.2018.00072

**Published:** 2018-04-24

**Authors:** Niklas Kästner, S. Helene Richter, Carina Bodden, Rupert Palme, Sylvia Kaiser, Norbert Sachser

**Affiliations:** ^1^Department of Behavioural Biology, University of Münster, Münster, Germany; ^2^Münster Graduate School of Evolution, University of Münster, Münster, Germany; ^3^Department of Biomedical Sciences, University of Veterinary Medicine, Vienna, Austria

**Keywords:** anxiety-like behavior, stress response, glucocorticoids, mice, social experience, adulthood, developmental plasticity

## Abstract

Social experiences can have profound effects on an individual’s level of anxiety. While various studies have addressed consequences of experiences of a specific type, e.g., social defeat, a recent study in mice investigated the impact of combinations of adverse and beneficial social experiences. Quite surprisingly, mice exposed to benefits during early life phases followed by escapable adversity in adulthood displayed lowest levels of anxiety, even compared to individuals having experienced throughout beneficial conditions. The present study aimed to elucidate whether this phenomenon is restricted to these specific life phases or whether it also exists when all these experiences are made in full adulthood. For this purpose, we compared anxiety-like behavior and stress response of adult male mice exposed to escapable social defeat following beneficial social experiences to that of mice exposed to either throughout adverse or throughout beneficial conditions. More precisely, we performed three established behavioral paradigms measuring anxiety-like behavior and assessed corticosterone metabolites non-invasively via feces sampling. Interestingly, we found no effects of social experience on anxiety-like behavior. In contrast to that, the animals’ stress hormone levels were profoundly affected by current social conditions: escapable social defeat (adverse condition) led to an increase in corticosterone metabolite concentrations, whereas living with a female (beneficial condition) led to a decrease. Thus, on the one hand this study suggests the importance of the timing of social experience for affecting an individual’s level of anxiety. On the other hand, it demonstrates that anxiety and stress hormone levels can be affected separately by social experience during adulthood.

## Introduction

Anxiety is a fundamental emotion of all vertebrates that can increase an individual’s chance of survival in dangerous situations (Marks and Nesse, 1994). In its exaggerated forms, however, it can have detrimental effects and lead to psychopathology (e.g., Kessler et al., 2005). Concerning the development of anxiety, the social environment has emerged as a key factor (Sachser et al., 2013). Considerable knowledge in regard to effects of various social experiences has been gained from work in rodent models, as here it is possible to experimentally address consequences of specific types of experience. These can be as diverse as for example high or low levels of maternal care (Liu et al., 1997; Caldji et al., 1998; Meaney, 2001), agonistic social encounters (Rodgers and Cole, 1993; Jansen et al., 2010; Kloke et al., 2011) or sexual interaction (Rodríguez-Manzo et al., 1999; Aikey et al., 2002; Edinger and Frye, 2007; Kästner et al., 2015). In particular, adverse and stressful social experiences, i.e., events that lead to an increase of glucocorticoid concentrations, have been linked to enhanced anxiety (Blanchard et al., 2001; Barik et al., 2013). Still, the mechanisms concerning the relation between stress hormones and anxiety remain far from being understood (McEwen, 2013; Raglan et al., 2017).

So far, studies have mainly focused on consequences of experiences of a specific type, e.g., social defeat or sexual experience. However, throughout ontogeny an individual is exposed to a variety of social events, and not much is known about possible interactions between different social experiences. In fact, a lot of theories trying to explain differences in vulnerability to psychiatric diseases in humans rely on accumulation of adversity over the life time (e.g., McEwen and Stellar, 1993; McEwen, 2003) or interactions between beneficial and adverse experiences (e.g., Bateson et al., 2004; Schmidt, 2011). Against this background, a study in mice recently performed in our lab wanted to elucidate how combinations of different social experiences affect anxiety: anxiety-like behavior of male mice was assessed after they had experienced distinct life histories consisting of different adverse and/or beneficial social experiences (Bodden et al., 2015). “Adverse” in this context refers to experiences potentially reducing reproductive fitness, while “beneficial” refers to experiences potentially increasing reproductive fitness. More specifically, during the pre- and early postnatal phase mice either lived in a dangerous or safe social environment, during adolescence they experienced either repeated losing or repeated access to a receptive female, and during early adulthood they were either exposed to escapable social defeat or housed together with a female. Most strikingly, when comparing mice having experienced throughout adverse conditions (AA group) to those having experienced throughout beneficial conditions (BB group), there was no difference in terms of anxiety-like behavior or exploratory locomotion. The only life history group that stood out in terms of relatively low levels of anxiety-like behavior and high levels of exploratory locomotion comprised mice having experienced early benefits (safe social environment during the pre- and early postnatal phase and repeated interaction with a receptive female throughout adolescence) followed by escapable social defeat in early adulthood (BA group). Thus, escapable adversity resulted in relatively low levels of anxiety compared to the other experimental groups but only in animals that had experienced benefits during previous life stages (Bodden et al., 2015). This finding was confirmed in a follow-up study (Remmes et al., 2016). Additionally, a second follow-up study revealed corresponding differences in the expression of genes associated with anxiety and stress circuits (Bodden et al., 2017). Altogether, these results gave rise to the formulation of the “coping with challenge” hypothesis. It states that coping with adversity (i.e., escapable social defeat) leads to relatively low levels of anxiety when following beneficial experiences in early life stages, but not when following adverse experiences. Interestingly, this effect seems to be specific to anxiety-like behavior: fear extinction seems to be affected in a different way than anxiety-like behavior by combinations of adverse and beneficial social experience (Remmes et al., 2016). For the described effect on anxiety, we assume that the controllability of the adverse experience is crucial. Notably, while controllability has been shown before to eliminate the anxiogenic effect of a stressor (Korte et al., 1999), in this study it even led to lower levels of anxiety than throughout beneficial conditions. Interestingly, studies in primates (e.g., Parker et al., 2006) and humans (e.g., Seery et al., 2013; Jezova et al., 2016) support the view that some challenge during development can be an advantage compared to the complete absence of adversity, a phenomenon referred to as “stress inoculation” (Parker et al., 2006) or “cross-adaptation” (Jezova et al., 2016). In these studies, exposure to mild adversity made individuals more resilient to subsequent stressful situations. In a comparable manner, in our studies in mice, escapable social defeat in the BA group might have resulted in greater resilience and thus lower levels of anxiety in the novel environment during the tests on anxiety-like behavior. However, the intensity of the adverse experience as well as the possibility to cope with it seem to be of importance for the described effect: continuous uncontrollable adversity during the pre- and early postnatal phase as well as adolescence in the AA group did not seem to bring about any effect of “cross adaptation” or “stress inoculation” for subsequent stressful events.

While it is generally believed that sensitivity to environmental stimuli is greatest during the pre- and early postnatal phase as well as during adolescence (Spear, 2000; Champagne and Curley, 2005; Sachser et al., 2011, 2013), there is also evidence that social experiences can still affect behavior in full adulthood (Buwalda et al., 2005; Jansen et al., 2010). With regard to this, the present study aimed to elucidate whether the effect of escapable social defeat following beneficial social experience described in mice also exists when all these experiences are made in adulthood. For this purpose, fully adult male mice were provided with beneficial social experience followed by escapable adversity. Anxiety-like behavior of this group was then compared to that of two control groups: one having experienced two adverse phases and the other having experienced two beneficial phases. In respect of the “coping with challenge” hypothesis, we predicted the combination of beneficial experience followed by escapable adversity to lead to lower levels of anxiety compared to continuous adverse or beneficial experience. As the stressfulness of social experiences is believed to play a major role in mediating effects on behavior, we also expected differences in stress hormone levels.

## Materials and Methods

### Animals and Housing Conditions

The study comprised 36 male mice of the C57BL/6J inbred strain purchased from a professional breeder (Charles River Laboratories, Research Models and Services, Germany GmbH, Sulzfeld, Germany) at an age of 3–4 weeks. Upon arrival, individuals were kept in groups of 2–4 until postnatal day (PND) 56, from when on they were housed individually. Experiments started at PND 71, which can be considered full adulthood (Brust et al., 2015). From arrival until the beginning of the second experimental phase, during which they were housed in special cage systems (see “Phase 2” section), animals were housed in polycarbonate cages type III (37 cm × 21 cm × 15 cm) with wood shavings as bedding material (Allspan, Höveler GmbH and Co. KG, Langenfeld, Germany), a semi-transparent red plastic house (11.1 cm × 11.1 cm × 5.5 cm, Tecniplast Deutschland GmbH, Hohenpeißenberg, Germany), a wooden stick and tissue paper as nesting material. Standard mouse diet (Altromin 1324, Altromin GmbH, Lage, Germany) and tap water were available *ad libitum*. The colony room was maintained at a temperature of about 22°C, a relative humidity of about 50%, and a 12 h light-dark cycle (lights on at 12 p.m.). Cages were cleaned and new enrichment was provided on a fortnightly basis. In addition to the test animals, 17 single-housed adult male NMRI mice and 38 group-housed adult female C57BL/6J mice purchased from Charles River Laboratories or derived from the internal stock of the Department of Behavioural Biology were used to provide the experimental animals with social experiences.

All procedures complied with the regulations covering animal experimentation within the EU (European Communities Council DIRECTIVE 2010/63/EU). They were conducted in accordance with the institutional and national guidelines for the care and use of laboratory animals and approved by the local government authorities (Landesamt für Natur, Umwelt und Verbraucherschutz Nordrhein-Westfalen, LANUV NRW, reference number: 84–02.04.2015.A441).

### Experimental Design

**Figure 1:**
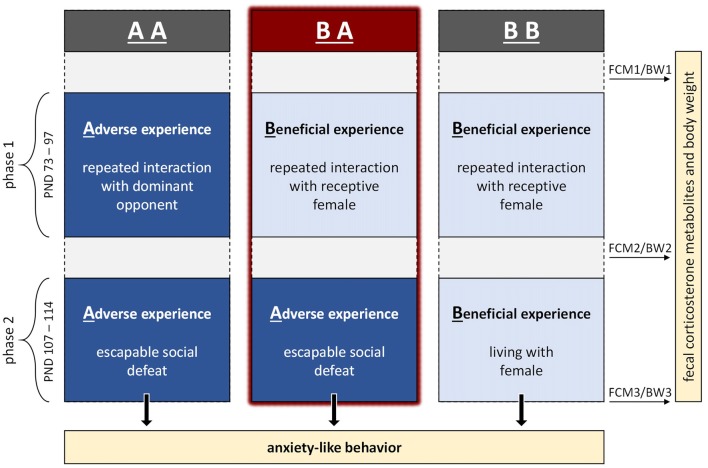
Experimental design. The experiment comprised two phases in adulthood, referred to as phase 1 and 2, during which male mice were provided with either adverse or beneficial social experiences. We compared mice having experienced benefits followed by escapable adversity (BA group) to two control groups: one having experienced throughout adverse conditions (AA group) and one having experienced throughout beneficial conditions (BB group). During the end of phase 2, mice were tested in three paradigms assessing anxiety-like behavior (PND 111–113). In addition to the behavioral tests, fecal corticosterone metabolites (FCM) and body weights (BW) were assessed on PND 71 (FCM1/BW1), 100 (FCM2/BW2) and 114 (FCM3/BW3).

The aim of the present study was to investigate, whether the combination of beneficial experience followed by escapable adversity in full adulthood leads to lower levels of anxiety compared to continuous adverse or beneficial experience. Please note that we were not mainly interested in how previous social experience affects following social experience, but in how combinations of social experiences affect levels of anxiety-like behavior. The experimental design (see Figure 1) was based on previous work describing such an effect in earlier life phases (Bodden et al., 2015; Remmes et al., 2016).

The experiment comprised two phases, referred to as phase 1 (PND 73–97) and phase 2 (PND 107–114), during which mice were provided with either adverse or beneficial social experiences. We compared mice having experienced benefits during phase 1 followed by adversity during phase 2 (BA group, *n* = 12) to two control groups: one having experienced adversity throughout phase 1 and 2 (AA group, *n* = 12) and one having experienced benefits throughout phase 1 and 2 (BB group, *n* = 12).

During the end of phase 2, mice were tested in three paradigms assessing anxiety-like behavior and exploratory locomotion (PND 111–113). In addition to these behavioral tests, fecal corticosterone metabolites (FCM) and body weights (BW) were assessed before phase 1, between phase 1 and 2 as well as at the end of phase 2.

The experiment was conducted in six replicates (*n* = 6 per replicate) delayed by 1 week, whereby experimental groups were balanced across replicates. As one mouse had to be excluded from the study, final sample sizes of individuals were: n_BA_ = 11, n_AA_ = n_BB_ = 12.

### Social Experiences

#### Phase 1

This phase comprised five encounters with a conspecific at an interval of 6 days (PND 73, 79, 85, 91, 97). Specifically, subjects were confronted each time either with an unfamiliar dominant male (adverse experience, **A**A group, see “Repeated Interaction with Dominant Opponent” section) or an unfamiliar female in pro-estrus or estrus (beneficial experience, **B**A and **B**B groups, see “Repeated Interaction with a Receptive Female” section). Encounters took place in the animals’ housing room during the dark phase under red light conditions. A transparent plastic cover was placed on the cage in which the interaction took place, allowing observation and at the same time preventing the mice from jumping out. While females and dominant males were re-used as interaction partners (maximum once per day), each experimental animal met each female/dominant male only once during the five encounters.

##### Repeated Interaction With Dominant Opponent

The experimental male was placed in the home cage (Makrolon type III) of an unfamiliar adult male of the aggressive NMRI strain (Navarro, 1997). A “losing experience” (see Jansen et al., 2010) was confirmed by an experienced observer (NK). The confrontation lasted for a maximum of 10 min, but was stopped early in cases of high aggression to prevent the animals from injury. The average number of attacks by the NMRI male during these confrontations was 4.3 ± 0.9 per minute (mean ± SD).

##### Repeated Interaction With a Receptive Female

An unfamiliar adult C57BL/6J female in pro-estrus or estrus was placed in the home cage of the experimental male and the animals could interact for 10 min. A socio-positive or sexual experience was confirmed by an experienced observer (NK). Estrous states of the females were assessed by microscopically examining vaginal smears, and the decision was made on the basis of the cytology (as described in Allen, 1922; Nelson et al., 1982; Byers et al., 2012; Kästner et al., 2017).

#### Phase 2

**Figure 2:**
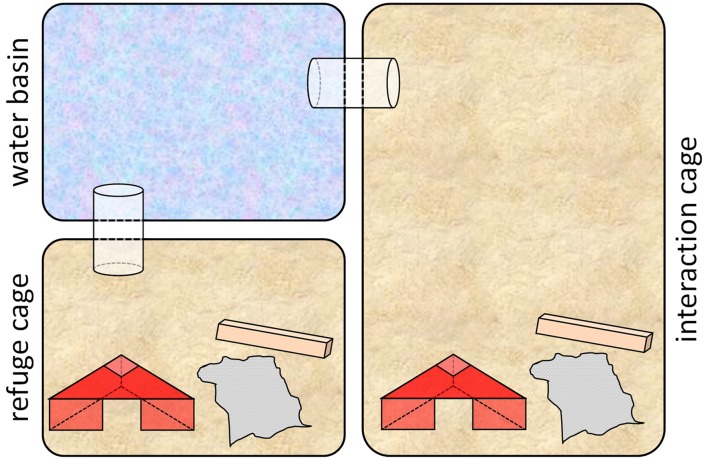
Cage system used in phase 2. The custom-made cage systems comprised an interaction cage (Makrolon type III with bedding and enrichment), a refuge cage (Makrolon type II with bedding and enrichment) and a water basin (Makrolon type II, filled with water to a height of approximately 3 cm; Figure modified after Meyer et al., 2016).

Throughout this phase (PND 107–PND 114), subjects were housed in special custom-made cage systems that comprised an interaction cage (Makrolon type III, with bedding and enrichment; food and water *ad libitum*), a refuge cage (Makrolon type II, with bedding and enrichment; food and water *ad libitum*) and a water basin (Makrolon type II, filled with water to a height of app. 3 cm; Meyer et al., 2016; see Figure 2). The water basin was connected to the interaction cage as well as to the refuge cage via plastic tunnels. Thus, to get from the interaction cage into the refuge cage, the water basin had to be crossed. Handling (as described in “Escapable Social Defeat” and “Living with a Female” sections) took place on PND 107, 108, 111, 112 and 113 between 8:30 a.m and 11:00 a.m, once per day for each experimental animal.

One day before the beginning of this phase, mice of all three groups were placed individually into the interaction cage of one cage system, while there was no water in the water basin. It was made sure that each individual had explored the whole set-up, including the refuge cage, before on the next day the first handling took place. Before the first handling, bedding and enrichment were renewed in each set up and either an aggressive NMRI male (adverse experience, A**A** and B**A** groups, “Escapable Social Defeat” section) or a female in pro-estrus or estrus (beneficial experience, B**B** group, “Living with a Female” section) were placed into the interaction cage of the setup, where they stayed throughout this phase.

##### Escapable Social Defeat

The experimental animal was placed into the interaction cage, i.e., to the NMRI male. As a response to the attacks of the NMRI mouse, it escaped via the water basin into the refuge cage. To prevent the NMRI mouse from following in any case, the tunnels were sealed by plastic inserts. These were only removed before the next handling on the next day, when the experimental animal was taken out of the refuge cage and again introduced into the interaction cage. Escape latencies were measured in minute intervals. Median escape latencies were below 3 min on the first day (PND 107) and below 1 min on the following days.

##### Living With a Female

The experimental animal was placed into the interaction cage, i.e., to the C57BL/6J female, where it stayed until the end of this phase. To correct for a possible handling effect, experimental animals of this condition were sham-handled on handling days. During these occasions they were given the possibility to escape via the water basin, what, however, never occurred.

### Anxiety-Like Behavior

Behavioral tests on anxiety-like behavior and exploratory locomotion were performed in the dark period between 12:00 a.m. and 4:30 p.m., i.e., during the animals’ active phase. Individual testing order was randomized on each test day and all equipment used for the tests was cleaned with 70% ethanol between subjects.

Tests were performed in a room separate from the subjects’ housing room, where they were transported in an empty Makrolon type II. Behavior was recorded by a camera (Logitech Webcam Pro 9000) and the animals’ movements were automatically analyzed by the video tracking system ANY-maze (version 4.75, Stoelting Co., Wood Dale, IL, USA).

#### Elevated Plus Maze

The Elevated Plus Maze (EPM; Pellow et al., 1985; Lister, 1987, 1990) was performed on PND 111. The maze consisted of four arms (30 cm × 5 cm each) and a central square (5 cm × 5 cm) in the shape of a plus and was elevated 50 cm above the ground by four wooden pillars. While two opposite arms were enclosed by 20 cm high wooden barriers (closed arms), the other two arms were surrounded only by a 0.4 cm high border to prevent the mice from falling off the maze (open arms). The wooden apparatus was painted light gray and the surface of the maze was covered by a gray PVC inlay. The illumination level in the center square was about 25 lux.

After having been transported to the testing room, individuals spent 1 min in an empty Makrolon type II cage and where then placed in the central square of the EPM with the head in direction of always the same closed arm. Now the experimenter left the room and the apparatus could be explored freely by the mouse for 5 min. Measures taken were relative number of open arm entries (open arm entries/(open arm entries + closed arm entries)) and relative time on open arms (time on open arms/(time on open + time on closed arms); anxiety-like behavior) as well as the total path traveled (exploratory locomotion).

#### Dark Light

The Dark Light (DL; Crawley and Goodwin, 1980) was performed on PND 112. The apparatus consisted of a modified Makrolon type III cage (37 cm × 21 cm × 15 cm). The dark compartment, created by black paint and dark plastic panels, made up one third of the cage and was connected to the remaining, illuminated part (light compartment) via a sliding door. The illumination level in the light compartment was about 35 lux.

After transport to the testing room, individuals spent 1 min in the dark compartment with the sliding door closed. Then the door was opened, the experimenter left the room and the apparatus could be explored freely by the mouse for 5 min. Measures taken were the latency to enter the light compartment and the time spent there (anxiety-like behavior) as well as the number of light compartment entries (exploratory locomotion).

#### Open Field

The Open Field (OF; Archer, 1973; Treit and Fundytus, 1988) was performed on PND 113. The apparatus consisted of a box made of white coated plywood with a square arena (80 cm × 80 cm) that was surrounded by walls (42 cm). The illumination level was set to about 35 lux. After transport to the testing room, mice were placed in a black cylinder (diameter: 11 cm, height: 20 cm) located in always the same corner of the field. After 1 min, the cylinder was lifted, the experimenter left the room and the mouse could freely explore the apparatus for 5 min. Measures taken were time in the center (defined as the area being located at least 20 cm distant from the walls) and number of center entries (anxiety-like behavior) as well as the total distance traveled (exploratory locomotion).

### Corticosterone Metabolites and Body Weight

Feces samples were collected to assess adrenocortical activity via measuring FCM. Additionally, BW were determined. FCM and BW measurement took place on three time points: before the beginning of phase 1 (PND 71, referred to as FCM1/BW1), between phase 1 and 2 (PND 100, referred to as FCM2/BW2) and at the end of phase 2 (PND 114, referred to as FCM3/BW3). We took feces samples not to obtain acute reaction values to the experimental procedures (e.g., interaction during phase 1 or handling during phase 2) but to measure the animals’ baseline levels of adrenocortical activity. During FCM3 we aimed to assess the effects of the current social environment (beneficial vs. adverse) on stress hormone levels in temporal proximity to the tests on anxiety-like behavior. FCM2 was assessed as an additional reference value, as we could not exclude changes in baseline adrenocortical activity due to experiences in phase 1.

The feces sampling procedure started directly after weighing between 12:30 p.m and 1:15 p.m. Mice were placed singly in a standard Makrolon cage III with a thin layer of bedding material, a paper towel and a red plastic house with food and water available *ad libitum* (see “Animals and Housing Conditions” section). After 3 h they were set back into their respective home cage and all feces produced during this time were collected and frozen at −20°C. Feces samples were then dried and homogenized, and aliquots of 0.05 g were extracted with 1 ml of 80% methanol (Palme et al., 2013). Afterwards, samples were analyzed using a 5α-pregnane-3β, 11β, 21-triol-20-one enzyme immunoassay, that was established and validated to measure corticosterone metabolites in mice (for details see Touma et al., 2003, 2004). Intra- and inter-assay coefficients of variation were below 10% and 12%, respectively. Sensitivity of the EIA was 1.7 ng/0.05 g.

### Statistical Analyses

Data were analyzed using general linear models. To meet the assumptions of parametric analysis, residuals were examined graphically for homoscedasticity and outliers, and using the Lilliefors corrected Kolmogorov–Smirnov test for normal distribution. When necessary, raw data were transformed using inverse (FCM) or logarithmic transformation (DL latency).

Behavioral data were analyzed using Univariate analysis of variance (ANOVA) with “experience” (BA, AA, BB) as fixed between-subjects factor. BW and corticosterone metabolite data were analyzed using repeated measures ANOVA with within-subjects factor “time” (FCM1, FCM2, FCM3 or BW1, BW2, BW3) and fixed between-subject factor “experience” as well as the interaction of “experience” and “time”. As Mauchley’s test of sphericity indicated no sphericity concerning the BW data, the Greenhouse-Geisser correction was applied. To present the magnitude of the reported effects in a standardized metric, effect sizes were calculated as partial eta squared ($\eta_{\text{p}}^{2}$; Lakens, 2013). In cases of significant main or interaction effects, Bonferroni-adjusted *post hoc* comparisons were conducted.

Differences were considered to be significant at *p* < 0.05 and statistical tests were run using the software package SPSS (IBM, v. 22 for Windows).

## Results

### Anxiety-Like Behavior

**Figure 3:**
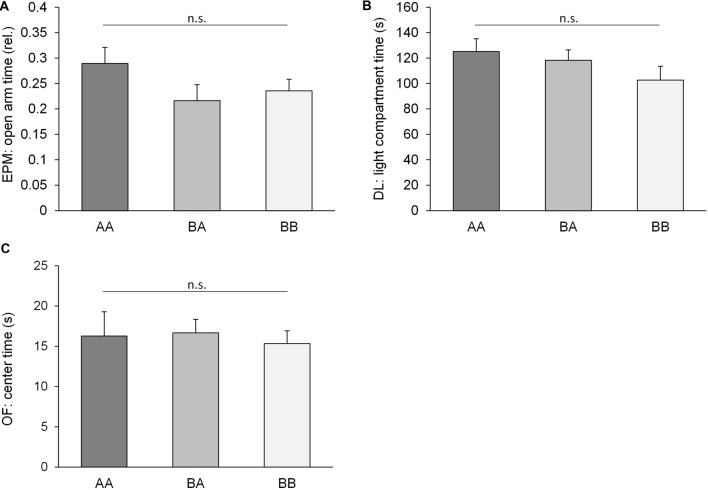
Anxiety-like behavior. Behavioral performance of adult male mice having experienced either benefits followed by escapable adversity (BA group) or throughout adverse (AA) or beneficial conditions (BB), respectively. Data are presented as means + SEM. Statistics: analysis of variance (ANOVA); sample sizes: n_AA_ = n_BB_ = 12, n_BA_ = 11; n.s. = *p* ≥ 0.05. **(A)** Elevated Plus Maze (EPM): rel. time on open arms; **(B)** Dark Light (DL): time in light compartment; **(C)** Open Field (OF) center time.

Strikingly, irrespective of profound differences in previous social experiences (AA: throughout adversity, BA: benefits followed by adversity, BB: throughout benefits), mice of the three groups did not differ significantly concerning anxiety-like behavior in any of the six parameters measured in three tests (EPM, rel. open arm entries: *F*_(2,32)_ = 2.282, *p* = 0.141; $\eta_{\text{p}}^{2}$ = 0.115; EPM, rel. open arm time, *F*_(2,32)_ = 1.599, *p* = 0.218, $\eta_{\text{p}}^{2}$ = 0.091; DL, light compartment latency: *F*_(2,32)_ = 0.818, *p* = 0.450, $\eta_{\text{p}}^{2}$ = 0.049; DL, light compartment time: *F*_(2,32)_ = 1.581, *p* = 0.221, $\eta_{\text{p}}^{2}$ = 0.090; OF, center entries: *F*_(2,32)_ = 0.601, *p* = 0.554, $\eta_{\text{p}}^{2}$ = 0.036; OF, center time: *F*_(2,32)_ = 0.086, *p* = 0.918, $\eta_{\text{p}}^{2}$ = 0.005; Figure 3). Similarly, there was no significant difference concerning exploratory locomotion (EPM, total path traveled: *F*_(2,32)_ = 0.500, *p* = 0.611, $\eta_{\text{p}}^{2}$ = 0.030; DL, light compartment entries, *F*_(2,32)_ = 1.960, *p* = 0.157, $\eta_{\text{p}}^{2}$ = 0.109; OF, total path traveled: *F*_(2,32)_ = 0.675, *p* = 0.516, $\eta_{\text{p}}^{2}$ = 0.040).

### Corticosterone Metabolites and Body Weight

#### Fecal Corticosterone Metabolites

Concerning FCM concentrations, statistical analysis revealed a significant main effect of time (*F*_(2,64)_ = 3.249, *p* = 0.045, $\eta_{\text{p}}^{2}$ = 0.092) as well as a significant time by experience interaction (*F*_(4,64)_ = 7.602, *p* < 0.001, $\eta_{\text{p}}^{2}$ = 0.322), while there was no significant main effect of experience (*F*_(2,32)_ = 0.020, *p* = 0.980, 0.001).

**Figure 4:**
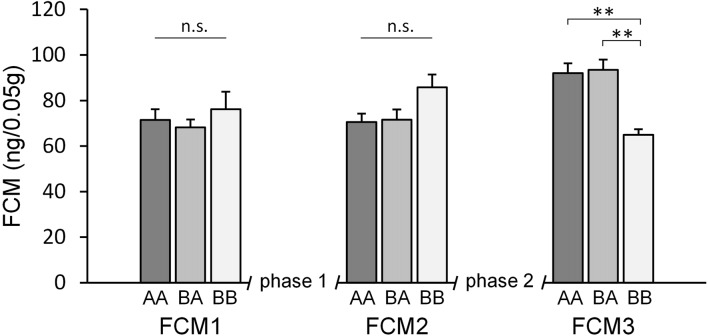
Fecal corticosterone metabolites (FCM). Adult male mice were provided with two experimental phases (phase 1 and 2), during which they either experienced benefits followed by escapable adversity (BA group) or throughout adverse (AA) or beneficial conditions (BB), respectively. FCM concentrations were assessed before phase 1 (FCM1, PND 71), between phase 1 and 2 (FCM2, PND 100) and at the end of phase 2 (FCM3, PND 114). There was a significant effect of time as well as a significant interaction between time and experience. Data are presented as means + SEM. Statistics: repeated measures ANOVA; *post hoc*: Bonferroni; sample sizes: n_BA_ = 11, n_AA_ = n_BB_ = 12; **p < 0.01, n.s. = *p* ≥ 0.05.

*Post hoc* analysis (see Table 1) revealed that groups did not differ significantly at the beginning of the experiments (FCM1; *p* > 0.679) as well as between phases 1 and 2 (FCM2; *p* > 0.128; Figure 4). At the end of the experiments (FCM 3), however, FCM concentrations were significantly lower in BB mice compared to both AA (*p* = 0.006) and BA mice (*p* = 0.002). This difference resulted from a change in FCM concentrations depending on social experience during phase 2 (FCM2 vs. FCM3): escapable social defeat led to a significant increase in FCM concentrations (AA: *p* = 0.018; BA: *p* = 0.005), whereas living with a female led to a significant decrease (BB: *p* = 0.002).

**Table 1:** Fecal corticosterone metabolites (FCM): *post hoc* comparisons.

Between time points across groups	Across groups		
	FCM1 vs. FCM2	FCM2 vs. FCM3	FCM1 vs. FCM3
	*p* = 0.906	*p* = 0.365	*p* = 0.061
Between groups within time points	FCM1		
	AA vs. BA	AA vs. BB	BA vs. BB
	*p* = 1.000	*p* = 1.000	*p* = 0.679
	FCM2		
	AA vs. BA	AA vs. BB	BA vs. BB
	*p* = 1.000	*p* = 0.128	*p* = 0.146
	FCM3		
	AA vs. BA	AA vs. BB	BA vs. BB
	*p* = 1.000	***p* = 0.006**	***p* = 0.002**
Between time points within groups	AA		
	FCM1 vs. FCM2	FCM2 vs. FCM3	FCM1 vs. FCM3
	*p* = 1.000	***p* = 0.018**	***p* = 0.048**
	BA		
	FCM1 vs. FCM2	FCM2 vs. FCM3	FCM1 vs. FCM3
	*p* = 1.000	***p* = 0.005**	***p* = 0.005**
	BB		
	FCM1 vs. FCM2	FCM2 vs. FCM3	FCM1 vs. FCM3
	*p* = 0.512	***p* = 0.002**	*p* = 0.217

*Repeated measures ANOVA revealed a significant main effect of time (*post hoc* test: pairwise comparisons between time points across groups) as well as a significant time by experience interaction (*post hoc* test: pairwise comparisons between groups within time points and between time points within groups). Statistics: Bonferroni *post hoc* adjustment following repeated measures ANOVA; sample sizes: n_BA_ = 11, n_AA_ = n_BB_ = 12; bold numbers: *p* < 0.05*.

### Body Weight

There was a significant main effect of time (*F*_(1.671,53.475)_ = 163.194, *p* < 0.001, $\eta_{\text{p}}^{2}$ = 0.836) and experience (*F*_(2,32)_ = 6.675, *p* = 0.004, $\eta_{\text{p}}^{2}$ = 0.294) on BW as well as a significant time by experience interaction (*F*_(3.342,53.475)_ = 9.751, *p* < 0.001, $\eta_{\text{p}}^{2}$ = 0.379).

**Figure 5:**
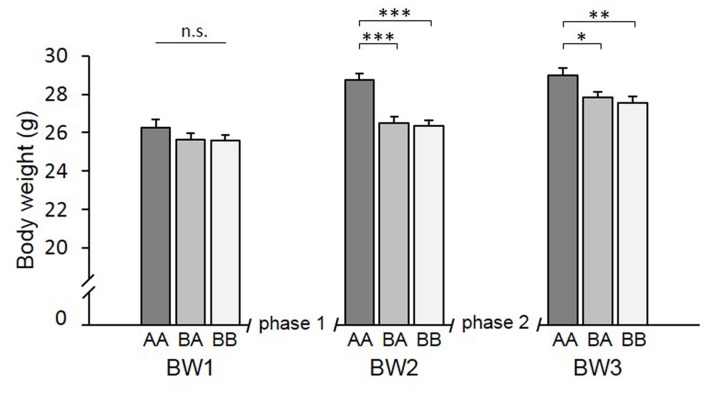
Body weight (BW). Adult male mice were provided with two experimental phases (phase 1 and 2), during which they either experienced benefits followed by escapable adversity (BA group) or throughout adverse (AA) or beneficial conditions (BB), respectively. BW was assessed before phase 1 (BW1, PND 71), between phase 1 and 2 (BW2, PND 100) and at the end of phase 2 (BW3, PND 114). There was a significant effect of time and experience as well as an interaction between these two factors. Data are presented as means ± SEM. Statistics: repeated measures ANOVA; *post hoc*: Bonferroni; sample sizes: n_BA_ = 11, n_AA_ = n_BB_ = 12; *p < 0.05, **p < 0.01, ***p < 0.001, n.s. = *p* ≥ 0.05.

*Post hoc* analysis (see Table 2) revealed that groups did not differ significantly at the beginning of the experiments (BW1; *p* > 0.752; Figure 5). After phase 1 (BW2), however, AA mice were significantly heavier than both BA (*p* < 0.001) and BB mice (*p* < 0.001). This was due to a higher BW gain in AA mice that experienced repeated losing during this phase. This difference in BW was still present at the end of phase 2 (BW3, AA vs. BA: *p* = 0.031, AA vs. BB: *p* = 0.005; AA vs. BA: *p* = 1.000).

**Table 2:** Body weight (BW): *post hoc* comparisons.

Between time points across groups	Across groups		
	BW1 vs. BW2	BW2 vs. BW3	BW1 vs. BW3
	***p* < 0.001**	***p* < 0.001**	***p* < 0.001**
Between groups across time points	Across time points		
	AA vs. BA	AA vs. BB	BA vs. BB
	***p* = 0.019**	***p* = 0.006**	*p* = 1.000
Between groups within time points	BW1		
	AA vs. BA	AA vs. BB	BA vs. BB
	*p* = 0.886	*p* = 0.752	*p* = 1.000
	BW2		
	AA vs. BA	AA vs. BB	BA vs. BB
	***p* < 0.001**	***p* < 0.001**	*p* = 1.000
	BW3		
	AA vs. BA	AA vs. BB	BA vs. BB
	***p* = 0.031**	***p* = 0.005**	*p* = 1.000
Between time points within groups	AA		
	BW1 vs. BW2	BW2 vs. BW3	BW1 vs. BW3
	***p* < 0.001**	*p* = 0.344	***p* < 0.001**
	BA		
	BW1 vs. BW2	BW2 vs. BW3	BW1 vs. BW3
	***p* = 0.003**	***p* < 0.001**	***p* < 0.001**
	BB		
	BW1 vs. BW2	BW2 vs. BW3	BW1 vs. BW3
	***p* = 0.008**	***p* < 0.001**	***p* < 0.001**

*Repeated measures ANOVA revealed a significant main effect of time (*post hoc* test: pairwise comparisons between time points across groups), a significant main effect of experience (*post hoc* test: pairwise comparisons between groups across time points) as well as a significant time by experience interaction (*post hoc* test: pairwise comparisons between groups within time points and between time points within groups). Statistics: Bonferroni *post hoc* adjustment following repeated measures ANOVA; sample sizes: n_BA_ = 11, n_AA_ = n_BB_ = 12; bold numbers: *p* < 0.05*.

## Discussion

The main finding of this study was: different combinations of adverse and beneficial social experiences in adulthood did not lead to significant differences in anxiety-like behavior. On the other hand, stress hormone levels were significantly higher in male mice having recently experienced escapable defeat compared to mice housed together with a female.

### Anxiety-Like Behavior

In previous studies, we have shown escapable social defeat in early adulthood to result in relatively low levels of anxiety, if mice had experienced benefits during early life, i.e., in the pre- and early postnatal period as well as adolescence (designated as “coping with challenge effect”; Remmes et al., 2016; Bodden et al., 2015). The major aim of the present study was to elucidate, whether this specific combination of experiences—benefits followed by escapable adversity—is sufficient for the occurrence of the “coping with challenge” effect or whether the phases of life during which these experiences are made are also important. More specifically: is it necessary that beneficial experiences are already made during early life? To answer this question, we investigated the effect of combinations of benefits and adversity with *all* experiences made in adulthood. Yet, in contrast to our expectation, anxiety-like behavior and exploratory locomotion of BA mice (benefits + adversity) did not differ significantly from that of either AA (adversity + adversity) or BB mice (benefits + benefits). Importantly, as we have not tested mice before and after the experiences, we cannot state that there was no effect on anxiety-like behavior at all. However, as AA, BA and BB mice did not differ significantly in any of the performed tests, we can conclude that the different combinations of experiences did not affect anxiety-like behavior differentially. Based on this and the fact that these social experiences did so when made in earlier life phases, we hypothesize that not only the combination and valence of experiences but also the life phases during which these experiences occur are indeed important for the described effect on anxiety. How can we explain this?

During the prenatal and postnatal phase before weaning, the brain is maturing and highly plastic (Rice and Barone, 2000). Also adolescence, the transition from infancy to adulthood, is marked by profound changes in brain architecture such as the remodeling of the prefrontal cortex (Spear, 2000; Paus, 2005; Steinberg, 2005). Social experiences during these phases have been shown to considerably affect the later behavioral profile of rodents (as reviewed in Champagne and Curley, 2005; Sachser et al., 2013). These effects comprise differences in social behavior (adolescence: Zimmermann et al., 2017; pre- and early post-natal phase: Kaiser et al., 2003; Kaiser and Sachser, 2001) as well as anxiety-like behavior (adolescence: Meyer et al., 2016; pre- and early post-natal phase: Liu et al., 1997; Caldji et al., 1998; Meaney, 2001). In adulthood, on the other hand, developmental plasticity is lower due to progressed brain maturation (Rice and Barone, 2000). Despite this, however, it has been shown that social experience can still modulate emotional behavior and underlying patterns of gene expression in adult rodents (Buwalda et al., 2005; Jansen et al., 2010; Bodden et al., 2017). In fact, also in the mentioned studies on the “coping with challenge” effect escapable adversity was experienced in early adulthood (Bodden et al., 2015, 2017; Remmes et al., 2016). The results of the present investigation in combination with our previous findings, however, suggest that for the occurrence of the “coping with challenge” effect specific social experiences have to be made already during early phases of life when brain circuits are highly plastic.

A further issue that should be addressed is that studies in mice and rats have indeed shown that un-controllable social defeat in adulthood can result in higher levels of anxiety-like behavior (Buwalda et al., 2005; Jansen et al., 2010). Yet, in these studies, effects of the experience have been found in close temporal proximity to the event. In our study, when the tests on anxiety-like behavior were performed, the last event of the first experience phase (social defeat vs. interaction with a female) was already 14 days ago. This might explain that next to no effects of the combinations of experiences, we did also not find an effect of the first experience (i.e., higher anxiety in AA mice compared to BA and BB mice).

### Corticosterone Metabolites and Body Weight

Unlike anxiety-like behavior, the animals’ stress hormone levels were profoundly affected by the current social environment: escapable social defeat led to an increase in FCM concentrations, whereas living with a female led to a decrease. While before phase 2 FCM concentrations did not differ between the groups, at the end of phase 2 they were significantly higher in AA and BA mice than in BB mice. This is in line with previous studies that found higher concentrations of glucocorticoids in defeated or subordinate animals (Sachser and Lick, 1989; Barik et al., 2013; Iñiguez et al., 2014; Bodden et al., 2015). One should not be surprised that FCM concentrations did not differ between the groups after phase 1, as during this phase experiences were made at considerable intervals and samples were taken three days after the last encounter.

Experiences that lead to an increase of glucocorticoid concentrations often also lead to an increase in anxiety (Blanchard et al., 2001). For example, social defeat in mice has been shown to result in higher corticosterone levels as well as higher levels of anxiety-like behavior (Jansen et al., 2010; Barik et al., 2013). Remarkably, in our study this was not the case: while the current social environment resulted in higher FCM concentrations in AA and BA mice than in BB mice, this difference was not reflected in the animals’ anxiety-like behavior. This finding highlights the fact that glucocorticoid levels and anxiety circuits can also be differently affected by social factors. This phenomenon is also known from other contexts, for example sexual behavior: while sexual experience decreases anxiety-like behavior in males (Rodríguez-Manzo et al., 1999; Aikey et al., 2002; Edinger and Frye, 2007; Kästner et al., 2015), it has been shown to result in increased corticosterone concentrations (Buwalda et al., 2012).

Concerning BW development, the main result was that repeated losing during phase 1 led to a significantly higher weight gain that persisted until the end of the experiment. At first glance, this might seem surprising, as social defeat has often been shown to result in a decrease in BW (Sachser, 1993; Tamashiro et al., 2007). However, the direction of the effect seems to depend on the intensity of the experience. In phase 1, experimental animals experienced five interactions with a dominant opponent at an interval of 6 days, i.e., at a low intensity. Actually, several studies in rodents show that mild social defeat can result in an increase in BW (Bartolomucci et al., 2004; Nestler, 2012; Bodden et al., 2015; Remmes et al., 2016). In fact, although in a different species and context it has been shown that higher BW gain can be an adaptive response to adverse conditions: a study in red squirrels showed a higher growth rate in offspring of mothers exposed to cues of high population density (Dantzer et al., 2013). During phase 2 weight gain still seemed to depend on experience during phase 1: AA mice did not gain weight but BA and BB mice did. Whether this was due to the already higher BW of AA mice after phase 1 (i.e., a ceiling effect) or due to other reasons, however, cannot be concluded on the basis of our data.

## Conclusion

Although adult mice experienced distinct histories of beneficial and/or adverse social experience, they did not differ concerning anxiety-like behavior. This and the fact that these combinations of experiences did affect anxiety when made in earlier life phases, suggests that the life phases in which social experiences are made are important for the shaping of the anxiety profile. While social experience did not result in differences in anxiety-like behavior and exploratory locomotion, however, it differently influenced FCM concentrations. This highlights that anxiety and stress hormone levels can be affected separately by social experience during adulthood.

## Author Contributions

SHR and NS conceived the study. CB, NK, SK, SHR and NS designed the experiments. NK performed the experiments and analyzed the data while NS and SHR supervised the project. RP determined and analyzed the hormonal data. NK wrote the initial draft of the manuscript and all other authors (CB, NK, SK, RP, SHR and NS) revised it critically for important intellectual content.

## Conflict of Interest Statement

The authors declare that the research was conducted in the absence of any commercial or financial relationships that could be construed as a potential conflict of interest.
